# A Computational Study Identifies HIV Progression-Related Genes Using mRMR and Shortest Path Tracing

**DOI:** 10.1371/journal.pone.0078057

**Published:** 2013-11-11

**Authors:** Chengcheng Ma, Xiao Dong, Rudong Li, Lei Liu

**Affiliations:** 1 Key Laboratory of Systems Biology, Shanghai Institutes for Biological Sciences, Chinese Academy of Sciences, Shanghai, P.R. China; 2 University of Chinese Academy of Sciences, Beijing, P.R. China; 3 Shanghai Center for Bioinformation Technology, Shanghai, P.R. China; 4 Institutes for Biomedical Sciences, Fudan University, Shanghai, P.R. China; George Mason University, United States of America

## Abstract

Since statistical relationships between HIV load and CD4+ T cell loss have been demonstrated to be weak, searching for host factors contributing to the pathogenesis of HIV infection becomes a key point for both understanding the disease pathology and developing treatments. We applied Maximum Relevance Minimum Redundancy (mRMR) algorithm to a set of microarray data generated from the CD4+ T cells of viremic non-progressors (VNPs) and rapid progressors (RPs) to identify host factors associated with the different responses to HIV infection. Using mRMR algorithm, 147 gene had been identified. Furthermore, we constructed a weighted molecular interaction network with the existing protein-protein interaction data from STRING database and identified 1331 genes on the shortest-paths among the genes identified with mRMR. Functional analysis shows that the functions relating to apoptosis play important roles during the pathogenesis of HIV infection. These results bring new insights of understanding HIV progression.

## Introduction

Many efforts have been devoted to better understanding the mechanism governing disease progression and non-progression during HIV infection. Besides the direct cytotoxic effect against CD4+ T cells caused by HIV, immune activation is widely accepted as a good predictor for disease progression [1], [2], [3], [4], [5]. Furthermore, some clinical studies using immunity suppressive drugs to suppress immune activation slowed down the diseases progression [6], [7]. However, the molecular mechanism underlying the immunopathogenesis remains obscure. To identify the host factors important for the HIV-1 pathogenesis and disease progression, high throughput techniques had been employed. Genome wide association studies revealed the protective effect against the virus of human leukocyte antigens (HLAs) including HLA-B*57:01, B*27:05 and risk alleles including HLA-B*35, Cw*07 [8], [9]. These studies further led to the finding of the protective effect of HLA-C [10] Transcriptome studies also gained important insights, regarding to interferon stimulated genes (ISGs), immune activation, cell cycle and cell death during the infection. People had conducted transcriptome researches for identifying factors affect the viral control and the speed of CD4+ T cell loss [11], [12]. A research with 137 HIV seroconverters, 16 elite controllers and 3 healthy blood donors attempted to identify some molecular factors associated with the viral control. More surprisingly, successful treatment made the transcriptome states of patients similar to the elite controllers and the HIV-negative donors [11]. Another study compared the transcriptoms of 6 viremic non-progressors (VNPs) and more than 20 rapid progressors (RPs). No significant result was found. Genes identified from the data of monkeys (*CASP1*, *CD38*, *LAG3*, *SOCS1*, *EEIFD*, and *TNFSF13B*) were deemed as the factors affecting the speed of disease progression [12].

Machine learning was recently proved to be an effective strategy for accurate classification of phenotypes based on transcriptome data (gene expression microarray) [13], [14], [15], [16], [17]. Among them, minimum redundancy – maximum relevance method (mRMR) is robust and represents a broad spectrum of characteristics [18], [19]. It was also developed to identify disease-related genes from expression profiles [18], [19].

Another useful informatics strategy for disease candidate gene identification is by known protein-protein interactions (PPIs). Since proteins not only function individually by themselves, but also co-function with their interaction partners; thus interaction partners of disease related genes are also important candidates for further disease casual studies. The STRING (Search Tool for the Retrieval of Interacting Genes) database is an online resource that provides PPI information by reporting from both prediction and experimental observations [20].

Here, we present a comprehensive informatics study based on transcriptional profiling of three different groups of HIV patients - rapid progressors (RPs), viremic controllers (ECs) and viremic nonprogressors (VNPs). We attempted to i) identify a gene set which can well classify the three groups, by using mRMR feature selection; ii) provide candidate casual genes for further experimental studies, by using shortest-path analysis of the above identified genes in a molecular interaction network contructed with the STRING data.

## Materials and Methods

### Gene expression profiling dataset of HIV patients

The dataset was from a research on HIV infection done by Rotger *et al.* [12]. In total, 78 chips were used in that research. We adopted data generated from CD4+ T cells, which contains 40 microarrays (8 elite controllers (ECs), 27 rapid progressors (RPs), 5 viremic non-progressors (VNPs)). Using the dataset alone, Rotger et al., didn't observe any differentially expressed genes. The data was downloaded from NCBI Gene Expression Omnibus (GEO) with the accession number of GSE28128. The expression profile was generated using the microarray Illumina HumanWG-6 v3.0 expression beadchip. Bead summary data was the output from Illumina's BeadStudio software without background correction. Genes declared as non-expressed (*P*>0.01) were excluded from further analysis. Data preprocessing, including quantile normalization and log2 transformation was completed in the Partek Genomics Suite package (Partek Inc.).

### Minimum redundancy – maximum relevance algorithm

Minimum redundancy – maximum relevance algorithm for selecting features (genes) was developed based on the idea to balance features’ ‘relevance’ to target (phenotype) and ‘redundancy’ between features [18]. Both relevance and redundancy are quantified using mutual information (MI). In this study, mRMR was realized using a R package ‘mRMRe’ [21], in which MI is estimated as,
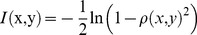
(1)where *I* and *ρ* represent the MI and the correlation coefficient between variables x and y, respectively.

Let y and X = {x_1_, …, x_n_} be the input variable (phenotype) and set of input features (genes), respectively. Given x_i_ as the feature with highest MI with the output variable, so the set of features, denoted by S is then initialized with x_i_.

In the second step, the feature x_j_ with the best balance between highest relevance and lowest redundancy was added to S. It is achieved by maximizing the score q as follows,
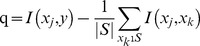
(2)The second step is repeat until a desired solution length has been reached.

### Prediction engine

In our analysis, predicted phenotype of an individual was estimated in three ways: the phenotype of its nearest neighbor; the most phenotype of its 5 nearest neighbor; the phenotype of its nearest clustering center of each phenotype group (Table S1). Distance between two individuals were calculated according to Chou et al.'s studies [22], [23], as follows,
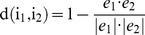
(3)where d refers to the distance, i_1_ and i_2_ represent two samples, and e_1_ and e_2_ are vectors of selected features (expression levels of selected genes) of the corresponding individual.

### Validation and Incremental Feature Selection

Jackknife validation was used to estimate the prediction accuracy of the selected features, considering its better performance and applicability to the dataset compared with other validation methods such as independent data set test and subsampling test [22], [24], [25]. Its idea is as follows. Given x samples with known outcome variable (phenotype) and n selected features (expression levels of genes), for each sample, we compared its known outcome variable with an estimated outcome variable, and the estimation was performed based on the rest x - 1 samples. The accuracy is defined using the following equation,
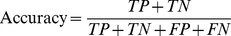
(4)where TP, TN, FP and FN represents the numbers of true positives, true negatives, false positives and false negatives, separately.

Incremental Feature Selection (IFS) was used to determine the number of prediction features. For n = 1 to 800 required number of features, each feature set was determined using mRMR and its prediction accuracy was estimated using Jackknife validation. The set with best prediction accuracy and smallest feature number was regard as final feature set. In this study, a set with 147 genes was chosen and its prediction accuracy is 0.8049.

### Shortest path identification in STRING PPI network

The background weighted PPI network was constructed using data from STRING database (version 9.1) (http://string-db.org) [20]. Weights of edges in the PPI network present confidence of the PPI ranging from 1 to 999. The Dijkstra's algorithm implemented in a R package ‘igraph’ [26], [27], was applied to identify shortest path between two pairs of proteins, and each of the protein corresponds to 86 protein coding genes of 147 mRMR-IFS identified genes. And the network of shortest paths was constructed using Cytoscape (version 3.0.1) [28].

### Pathway enrichment analysis

KEGG pathway enrichment and GO functional enrichment analysis were carried out using web service of DAVID tools (version 6.7) [29], [30]. Original enrichment *P* values as well as *Benjamin multiple test* corrected *P* values were estimated.

## Results

### A set of 147 genes presents the best accuracy for prediction of RPs, ECs and VNPs

**Figure 1:**
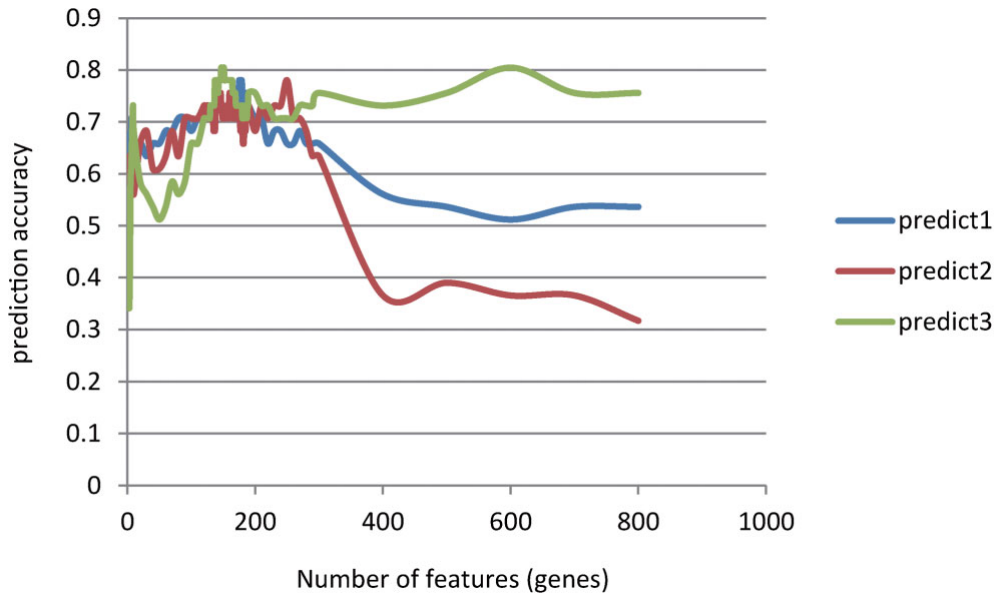
IFS curve to determine the number of features used in prediction. We used an IFS curve to determine the number of features finally used in mRMR selection. Prediction accuracy reached its maximum value when 147 genes were included. The ‘predict1’, ‘predict2’ and ‘predict3’ refer to the three prediction methods we used – a vote of the top five nearest neighbor, the first nearest neighbor and nearest clustering center of each phenotype group, seperately.

**Figure 2:**
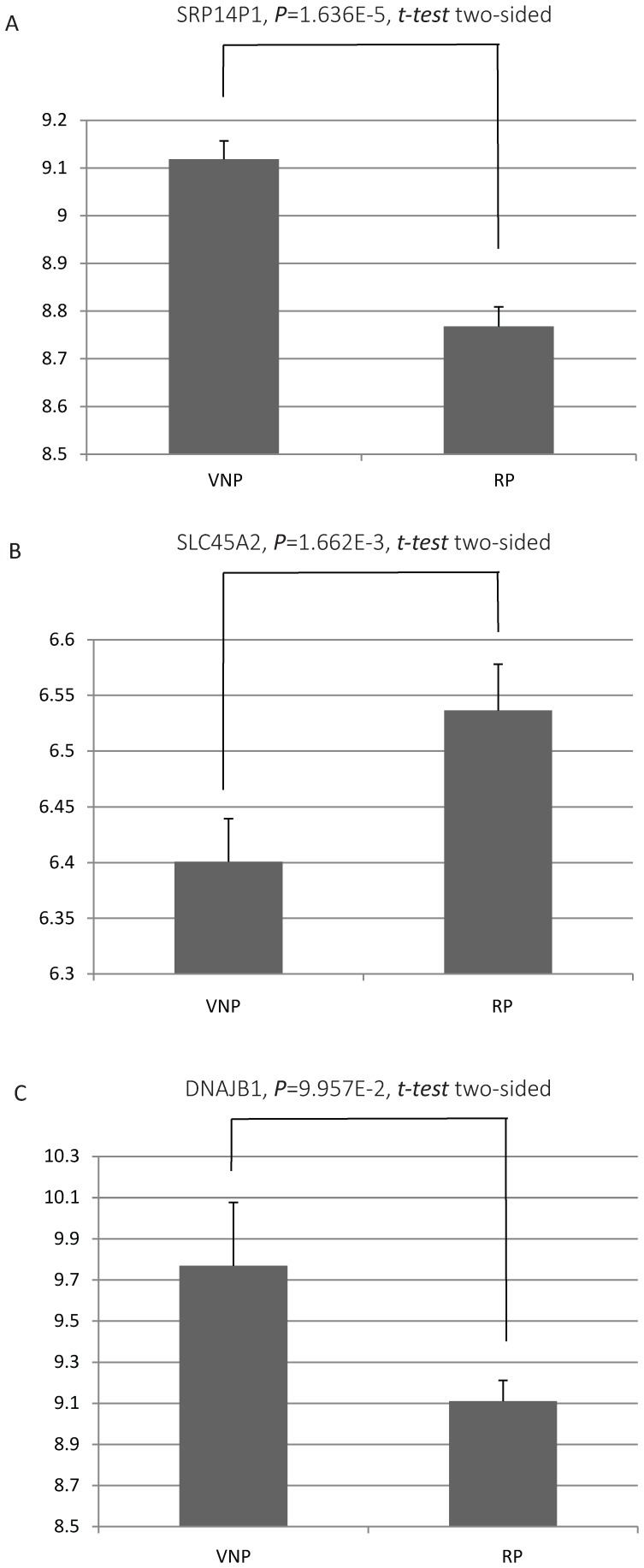
Expression differences of *SRP14P1*, *SLC45A2*, and *DNAJB1* between VNPs and RPs. This figure show the expression differences of *SRP14P1* (A), *SLC45A2* (B), and *DNAJB1* (C) between VNPs and RPs, separately. Error bars indicate standard errors.

Based on the outputs of mRMR, we tested the predictor with one feature (probes of gene expression array), two features, three features, etc., and the IFS result is provided in Figure 1. In the IFS curve, the X-axis is the number of probes used for classification, and the Y-axis is the prediction accuracies of the nearest neighbor algorithm evaluated by the Jackknife validation. The accuracy reaches its maximum value when 147 features were included, corresponding to 147 different genes (Table 1 for 20 genes identified with top mRMR scores and Table S2 for the full list). We discussed reported function of three genes in Table 1, *SRP14P1*, *SLC45A2*, and *DNAJB1*. Figure 2 A to C show their expression difference between VNP and RP. Although fold changes are not dramatic, there are significant difference between them (*P* value, 0.09957 to 1.636*10^−5^).

**Table 1:** Top 20 of the 147 genes by mRMR score.

Probe ID	Gene Symbol	mRMR score
ILMN_2211950	SRP14P1	0.374054816
ILMN_1908490	KRTAP24-1	0.189859156
ILMN_1685259	SLC45A2	0.187915652
ILMN_1667517	LOC643329	0.187764108
ILMN_1805778	RBM12B	0.182771664
ILMN_1766475	MGAT3	0.180021681
ILMN_1790650	C16orf63	0.179598512
ILMN_2393712	CTTN	0.177892746
ILMN_1731157	MYOZ1	0.174400886
ILMN_1662845	NBPF11	0.173616766
ILMN_1775304	DNAJB1	0.171712561
ILMN_1742846	DIRAS2	0.164352488
ILMN_1724271	LOC648976	0.162551286
ILMN_2215103	LOC644152	0.162538695
ILMN_1774617	NAB1	0.161792112
ILMN_1702009	SV2A	0.161418466
ILMN_1710092	ZBTB46	0.16034942
ILMN_1733799	LOC348262	0.157032268
ILMN_1770038	LAMA1	0.15612958
ILMN_1695397	LOC644151	0.153023869

### A PPI sub-network provides insights for the 147 genes on HIV infection

**Figure 3:**
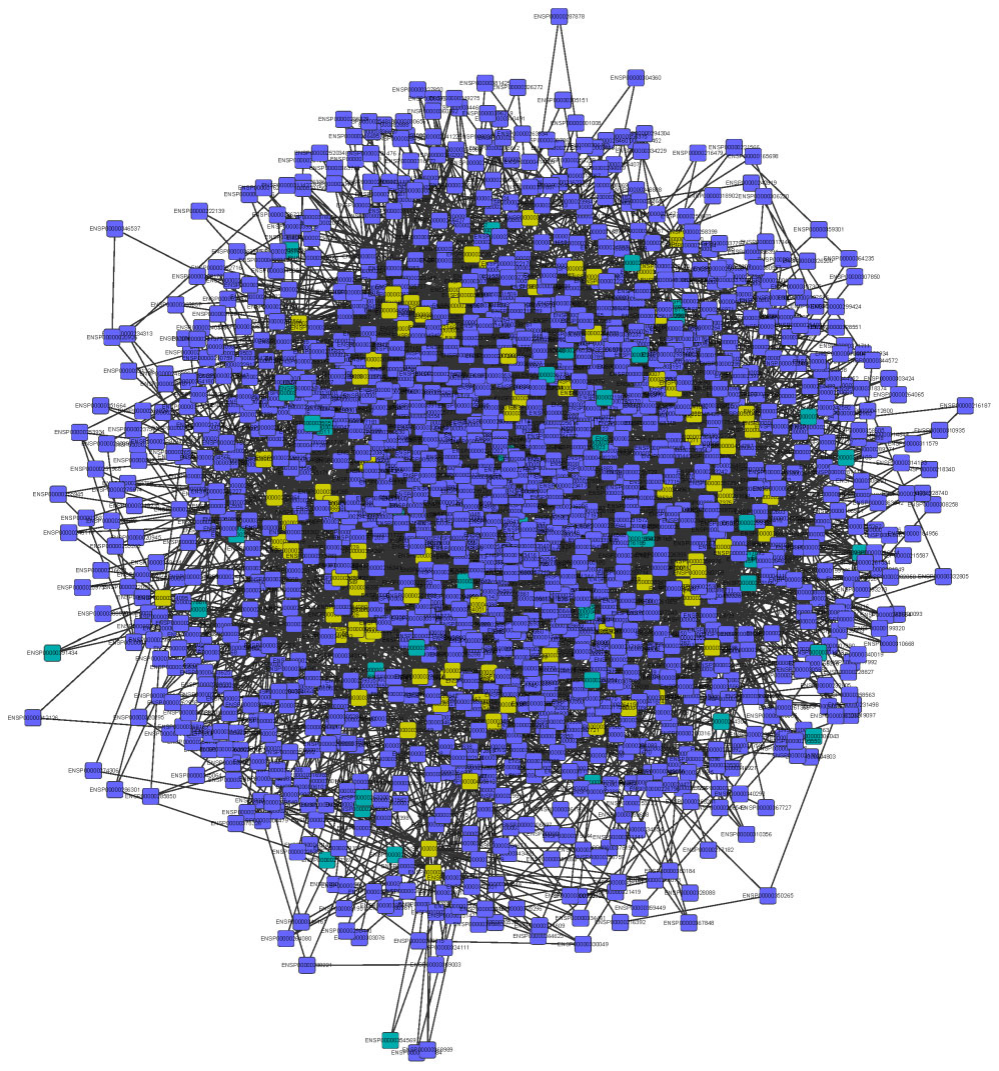
PPI network of shortest paths among 86 mRMR identified proteins. Shortest paths between each pair of the 86 mRMR selected proteins were identified in the STRING PPI network. Proteins are presented using their Ensemble IDs. Proteins in yellow are the 86 identified using mRMR; in blue and green are located only on shortest paths; in blue are annotated in Ensemble Biomart; and in green are not annotated in Ensemble Biomart.

Furthermore, we constructed an undirected graph with the PPI data from STRING [20]. Then we picked all pairs of any two genes from 86 protein coding genes of the 147 genes identified with mRMR as described above, and revealed the shortest path between these two proteins using the Dijkstra's algorithm [26]. We eventually obtained a total of a sub-network of STRING PPIs based on the shortest paths (Figure 3). There are a total of 4248 protein-protein interactions of 1331 proteins. Among the 1331 proteins, 1290 of their corresponding genes were annotated in the Ensemble Biomart database, and we ranked these genes according to their betweennesses (Table 2 for the top 20 and Table S3 for the full list). Among these 1290 genes, *UBE2K* has the largest betweenness of 66, meaning that there are at least 33 shortest paths going through this gene. Accordingly, UBE2K may play an important role in connecting the 66 candidate genes and hence may be related to the loss of CD4+ T cells, although we didn't find any previous reports about the effect of UBE2K on the loss of CD4+ T cells.

**Table 2:** Top 20 of the 1290 genes by betweenness in the shortest paths among the 86 mRMR identified genes.

Ensembl Gene ID	Ensembl Protein ID	Associated Gene Name	mRMR gene	betweenness
ENSG00000078140	ENSP00000261427	UBE2K	TRUE	66
ENSG00000147889	ENSP00000355153	CDKN2A	TRUE	65
ENSG00000196406	ENSP00000359546	SPANXD	TRUE	60
ENSG00000150991	ENSP00000344818	UBC	FALSE	56
ENSG00000198728	ENSP00000392466	LDB1	TRUE	55
ENSG00000049449	ENSP00000054950	RCN1	TRUE	54
ENSG00000087338	ENSP00000282570	GMCL1	FALSE	54
ENSG00000143632	ENSP00000355645	ACTA1	TRUE	54
ENSG00000178919	ENSP00000364265	FOXE1	TRUE	54
ENSG00000132002	ENSP00000254322	DNAJB1	TRUE	53
ENSG00000197894	ENSP00000296412	ADH5	TRUE	53
ENSG00000183808	ENSP00000382239	RBM12B	TRUE	53
ENSG00000141232	ENSP00000268957	TOB1	TRUE	52
ENSG00000116761	ENSP00000359976	CTH	TRUE	52
ENSG00000164125	ENSP00000377396	FAM198B	TRUE	50
ENSG00000185630	ENSP00000405890	PBX1	TRUE	48
ENSG00000137804	ENSP00000260359	NUSAP1	TRUE	46
ENSG00000115267	ENSP00000263642	IFIH1	TRUE	46
ENSG00000138777	ENSP00000343885	PPA2	TRUE	46
ENSG00000006283	ENSP00000352011	CACNA1G	TRUE	46

### Function and pathway enrichment of the identified genes

Using the functional annotation tool DAVID [29], [30], the Gene Ontology (GO) and KEGG pathway enrichment analysis were carried out for the 147 genes identified using mRMR as well as the 1290 genes on the shortest paths (Table S4). The results showed that these genes were significantly enriched in the GO modules of protein localization in organelle and SMAD protein nuclear translocation GO modules. In KEGG pathway enrichment analysis, no pathway was significantly enriched. These results suggested that protein location may play critical role in the pathogenesis during the chronic HIV infection.

We also analyzed the genes on the shortest paths among the genes identified by mRMR algorithm with GO and KEGG enrichment. In the GO enrichment analysis, many genes were significantly enriched in GO modules relating to negative regulation of apoptosis (Table S5). Early studies had shown the importance of apoptosis during the pathogenesis of HIV infection [31], [32]. Vulnerability to apoptosis is a hallmark character of CD4+ T cells due to immune activation and Tat protein [31], [33]. Our results supported that resistance to apoptosis might be an important mechanism of the VNPs to maintain their CD4+ T cell levels. In the KEGG enrichment results, nothing strongly relating to the high CD4+ T cell level was discovered (Table S6).

## Discussion

### Using mRMR to identify candidate gene set

We applied mRMR algorithm to study HIV progression related genes based on transcriptome data. The major difference between mRMR and another machine learning method is that mRMR considers redundancies among features as well as relevance between feature and target. Therefore, disease-related genes which shown less correlation in expression among themselves, are favored by the mRMR algorithm. This may be the theoretical reason that genes identified in our study represent various functional groups in GO annotations (Table S4).

### Result validation

In this study, we used a jackknife method to validate our result. In jackknife validation, one sample x_i_ is excluded from all the n samples X = {x_i_|i = 1,2,3 … n}. We predict its phenotype using the rest n-1 samples, and compared its predicted phenotype with the real one. The above step is repeated for all x_i_, i = 1,2,3 … n. In this way, prediction accuracy of X is considered as a parameter of validation. Previous studies also discussed the advantages of jackknife validation over another validation methods, such as subsampling test and independent data set test [22], [24], [25]. Jackknife validation can exclude “memory” effect as well as arbitrariness problem existed using the other two validation methods.

Although jackknife validation was proved to be useful, we also considered using an independent dataset of general HIV infection as an additional validation to our results. The independent dataset is composed of expression profiles of CD4 T cells from 127 HIV untreated samples and 8 normal controls. Using student t test, we observed that 46 genes out of the 147 mRMR selected genes differentially expressed under *P* value 0.05 [11]. We included the related results in Table S2.

### mRMR selected Genes

In the 147 genes optimizing the mRMR prediction, a series of SLC (*Solute Carrier Family*) genes, e.g. *SLC4A8* (mRMR score = 0.1096), *SLC22A10* (mRMR score = 0.1159), *SLC22A15* (mRMR score = 0.1314), *SLC25A1* (mRMR score = 0.1240), *SLC28A1* (mRMR score = 0.1260) and *SLC45A2* (mRMR score = 0.1879) are included. As SLC gene products are transmembrane proteins transporting/translocating substrates (e.g. inorganic or organic ions, sugars, nucleosides, etc.) into/out of cells [34], it can be fairly assumed that such genes have functional influences on HIV/AIDS-related processes.

In fact, polymorphisms (e.g. SNPs) of human SLC transporter genes have been found to be closely related to HIV infection or responses to treatments of HIV-infected patients. For example, the genotypes of *SLC11A1*, a proton-coupled metal ion transporter, have determining effects on mortality in HIV infection [35]; SNPs of loci in or near *SLC26A7*, a multifunctional anion exchanger linked to energy metabolism and immunoregulations, are strongly associated with upper trunk and arm subcutaneous adipose tissue (SAT) distribution in antiretroviral therapy (ARV)- treated HIV-infected patients [36]. Furthermore, literatures suggest that antiretroviral drugs such as efavirenz and nevirapine are substrates of a variety of SLCs and SNPs of them may influence highly active antiretroviral therapy (HAART) efficacy and AIDS-free survival [37], [38]. Thus it is no surprise that the SLC genes included in our results of mRMR (*4A8*, *22A10*, *22A15* - inorganic/organic ions transporters; *25A1* - mitochondrial carrier; *28A1* - nucleoside transporter; *45A2* - putative sugar transporter), which have similar functions to those in the literatures, may sustain similar functional roles and serve as good surrogates for prediction of HIV/AIDS outcomes.

Another gene among the 147 genes we noticed is *Mouse Double Minute 2 Homolog* (*MDM2*) (mRMR score = 0.1185). It is also known as E3 ubiquitin-protein ligase Mdm2, which is a negative regulator of p53 tumor suppressor [39]. Mdm2 inhibits p53 cell-cycle arrest and apoptic functions and the interaction with Mdm2 can also result in a large reduction in p53 protein levels through enhanced proteasome-dependent degradation. Endogenous levels of Mdm2 are sufficient to regulate p53 stability, and overexpression of Mdm2 can reduce the amount of endogenous p53. Mdm2 is also found to be important for lymphopoiesis through the inhibition of p53 [40], which makes it a potential factor regulating the CD4+ T cell counts during chronic HIV infection.

The importance of gene *DNAJB1* (*DnaJ homolog subfamily B member 1*, mRMR score = 0.1717,) to HIV/AIDS is self-evident, as experimental studies show that it is regulated by various subtypes of HIV (e.g. HIV-1 B, C and A/E) in dendritic cells (DCs), which are among the first targets of HIV infection and in turn play crucial roles in viral transmission to T cells and regulation of immune responses [41], [42]. Meanwhile, its gene product DNAJB1 (Hsp40), is essential for HIV-1 Nef-mediated enhancement of viral gene expression and replication, thus playing a key role in the virus life cycle [43], [44].

*SRP14P1* (scored the highest 0.374 by mRMR) is named as *Signal Recognition Particle 14 kDa (Homologous Alu RNA Binding Protein) Pseudogene 1*, which is rarely studied. *SRP14* is an Alu RNA binding protein. It regulates the expression of Alu elements, which is an important expression regulation element. This finding in our work suggested its potential role in affecting the pathogenesis of HIV infection.

Interferon response is of important function in HIV infections. But we didn't observe interferon stimulated genes. As suggested by Rotger et al. [11], during the course of HIV infection the interferon stimulated genes are stimulated by the virus. So it may explain that in our case, holding the viral load equal, the interferon stimulated genes are not identified.

## Conclusion

In the previous study [12], M. Rotger *et al.* endeavored to explore some molecular factors relating to the maintenance of CD4+ T cell level using the transcriptome derived from CD4+ T cells of VNPs and RPs. The results were not encouraging. Therefore, we made the attempt to discover something new from a different perspective. Although most genes found in our results did not closely relate to the different CD4+ T cell levels between the two groups of people, functions associated with apoptosis regulation had been identified. These results reflected the complexity of the mechanism governing the decline of CD4+ T cell count during HIV infection. To some extent, this could also be explained by the design of the study. The data were generated from a retrospective study, thus the factors mediating the decline of CD4+ T cells might function early among the RPs and returned to normal since the CD4+ T cells had already dropped to a low level. Taking that into consideration, perspective studies for further discoveries could become feasible.

## Supporting Information

Table S1 **Predictions of phenotype of each individuals using the 147 genes identified using mRMR.**
(TXT)

Table S2 **The 147 genes selected using mRMR.**
(TXT)

Table S3 **The 1290 genes in the shortest paths between the 86 mRMR identified genes.**
(TXT)

Table S4 **GO enrichment of the 147 mRMR identified genes.**
(TXT)

Table S5 **GO enrichment of the 1290 shortest path identified genes.**
(TXT)

Table S6 **KEGG pathway enrichment of the 1290 shortest path identified genes.**
(TXT)
